# Combining three‐dimensional fluoroscopy guide system with single‐use bronchoscopes for diagnosis of peripheral lesions: First two cases

**DOI:** 10.1002/rcr2.1113

**Published:** 2023-03-10

**Authors:** Filippo Patrucco, Andrea D'Alessio, Marco Brambilla, Piero Emilio Balbo

**Affiliations:** ^1^ Respiratory Diseases Unit, Medical Department Maggiore della Carità Hospital Novara Italy; ^2^ Translational Medicine Department University of Piemonte Orientale Novara Italy; ^3^ Medical Physics Department Maggiore della Carità Hospital Novara Italy

**Keywords:** 3D fluoroscopy, lung cancer, peripheral lesions, single‐use bronchoscopes, solitary pulmonary nodule

## Abstract

Pulmonary Peripheral Lesions (PPLs) diagnosis is usually performed using a guidance system in combination with bronchoscopes and diagnostic tools. We report two cases of PPLs sampling procedures combining the use of the single‐use bronchoscope Ambu aScope 5 Broncho and CIOS 3D Spin Mobile (Siemens Healthineers) fluoroscopy system. A 69‐year‐old‐female was found to have a lesion located in right B6 segment and a 73‐year‐old‐male with a mass in the upper right lobe. We used for both cases a single‐use bronchoscope to reach the correct area and the fluoroscopy system to guide peripheral transbronchial aspiration needle (TBNA) sampling. After the confirmation of the correct location of the TBNA tool, the sampling was performed. Rapid onsite evaluation (ROSE) confirmed the adequacy of the sample for molecular analysis and the final diagnosis. Thus, the use of ever‐new disposable bronchoscopes for sampling peripheral lesions is a viable alternative to reusable bronchoscopes for advanced bronchoscopy procedures.

## Graphical abstract

Pulmonary peripheral lesions (PPLs) diagnosis is usually performed using a guidance system that combines bronchoscopes and diagnostic tools. We report two cases of PPLs sampling procedures combining the use of aScope 5 single‐use bronchoscope and CIOS 3D Spin Mobile fluoroscopy system. The use of ever‐new disposable bronchoscopes for sampling peripheral lesions is a viable alternative to reusable bronchoscopes for advanced bronchoscopy procedures.

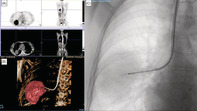

## INTRODUCTION

Pulmonary peripheral lesions (PPLs) diagnosis represents one of the most intriguing challenges for interventional pulmonologists. Although several devices, diagnostic tools and guidance systems have been developed to reach PPLs, diagnostic performances outside research studies remain non satisfactory.^1^ Fluoroscopy represents the most commonly used system worldwide, using a C‐arm or a biplane fluoroscope which allows the real‐time visualization of the sampling instrument and PPL, both in antero‐posterior and lateral views.^1^ The diagnostic yields are variable among studies (19%–83%) and are influenced by PPLs characteristics (size, location, presence of bronchus sign) as well as sampling tool used (forceps, TBNA needles).^1^ The CIOS 3D Spin Mobile system is a portable C‐arm, with a three‐dimensional (3D) fluoroscopy system that, rotates around the patient's chest by a ± 100°, generates a CT image allowing the operator to confirm the correct position of the tool before the sampling.^2^ This system partially overcomes the limitations of conventional fluoroscopy systems, permitting the correct visualization of the interrelationship between the bronchial path, in which the sampling tool is inserted, and lesion's shape, ensuring promising results in terms of diagnostic performances and complications.^2^

Single‐use bronchoscopes (SUBs) are typically used for diagnostic and therapeutic purposes (mainly bronchoalveolar lavage, removal of clots and mucous plugs) although more complex diagnostic procedures have been recently described: sampling of endobronchial lesions using forceps, endobronchial valve placement, thermal ablative therapy, cryotherapy, tumour debulking, foreign body retrieval, airway stent deployment, guidance for percutaneous tracheostomy procedure.^3^ Up until now, several technical characteristics of SUBs limited their role in PPLs diagnostics: the small diameter of working channel was not compatible with TBNA needles; the flexibility of the distal extremity with tools inserted would limit the manoeuvrability, especially in case of PPLs located in upper lobes; the concern that SUBs might not be robusts enough to allow them to withstand the stress of repeated sampling passes. Ambu® aScope™ 5 Broncho is the 5th generation of the Ambu single‐use bronchoscopes: it is designed for use with the diagnostic and therapeutic endotherapy instruments commonly used in the bronchoscopy suite such as biopsy forceps and needles, cytology brush, cryoprobes and high‐frequency tools. The combination of the bending angles, distal tip angulation range (195°/195°) and the flexibility of the insertion tube is designed to help access difficult‐to‐reach areas. According to a recent publication, the aScope 5 has more degrees of flexion/extension with tools compared to reusable flexible bronchoscopes (RBs). The manoeuvrability is further enhanced by 120° left/right rotation designed to make it easy to adjust the position of the tip to ease navigation and insertion of endotherapy instruments.^4^

We report two cases of PPLs sampling procedures combining the use of aScope 5 single‐use bronchoscope and CIOS 3D Spin Mobile fluoroscopy system.

## CASE REPORT

### Case 1

**FIGURE 1:**
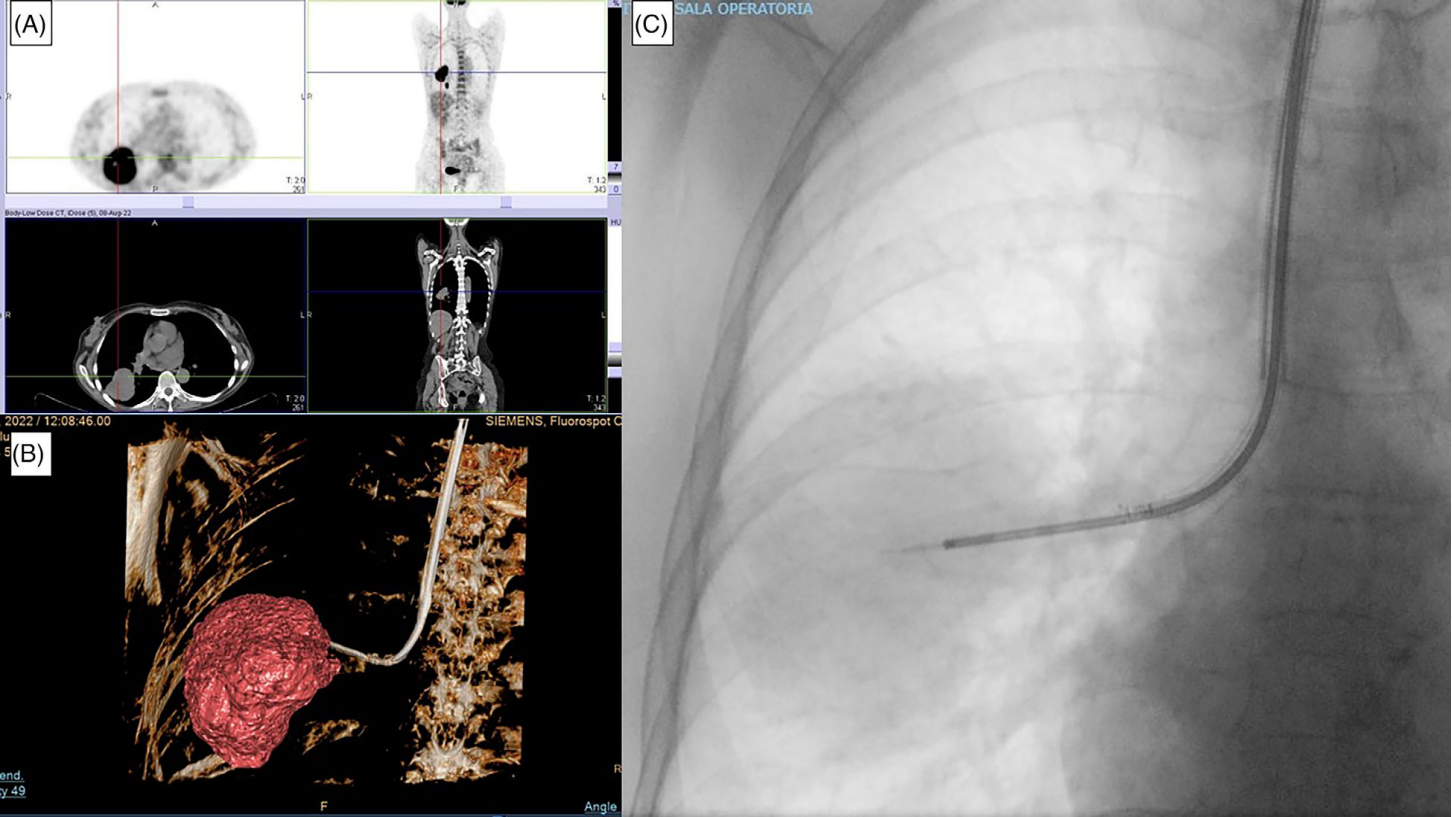
(A) 18F‐fluorodeoxyglucose (FDG) PET/CT uptake in correspondence to the right B6 segment; (B) aScope 5 Broncho HD 5.6/2.8 single‐use bronchoscope reaches the lesion guided by 3D fluoroscopy CIOS guidance system; C. fluoroscopic image of TBNA sampling of the lesion.

A 69‐year‐old female, active smoker, come to our attention after the onset of 8 kilograms of weight loss associated with dry cough. She performed a chest X‐ray and then a thorax CT scan revealing a pulmonary mass (54 × 44 × 55 mm) located in right B6 segment: the lesion presented a bronchus sign type A (bronchus reaches the inside of the target lesion) and infiltrates the fissure repassing it. The next positron emission tomography (PET) CT scan confirmed the high concentration of 18F‐FDG in correspondence with the lesion without suspected lymph nodes involvement (Figure 1A).

We performed the bronchoscopy in spontaneous breathing, in conscious sedation. We used aScope 5 Broncho HD 5.6/2.8 single‐use bronchoscope: endoscopically no lesions were detectable, so we used the CIOS system to guide peripheral TBNA sampling (21‐Gauge needle). After the confirmation of the correct location of the TBNA tool (Figure 1B, C), we proceeded with the sampling. ROSE confirmed that we reached the lesion (adequate material suspected for presence of neoplastic cells) so we completed the sampling with another three passes. No procedure‐related complications were recorded; the TBNA tool was easily introduced in the bronchoscope working channel without problems of manoeuvrability, with or without the needle inserted in; the bronchoscope was correctly operating after the procedure. Acquisition time and dosimetric index (air kerma‐area product, KAP) were respectively 79 s and 10.2 Gycm^2^. Final diagnosis was adenocarcinoma and the material obtained was adequate for molecular analysis.

### Case 2

**FIGURE 2:**
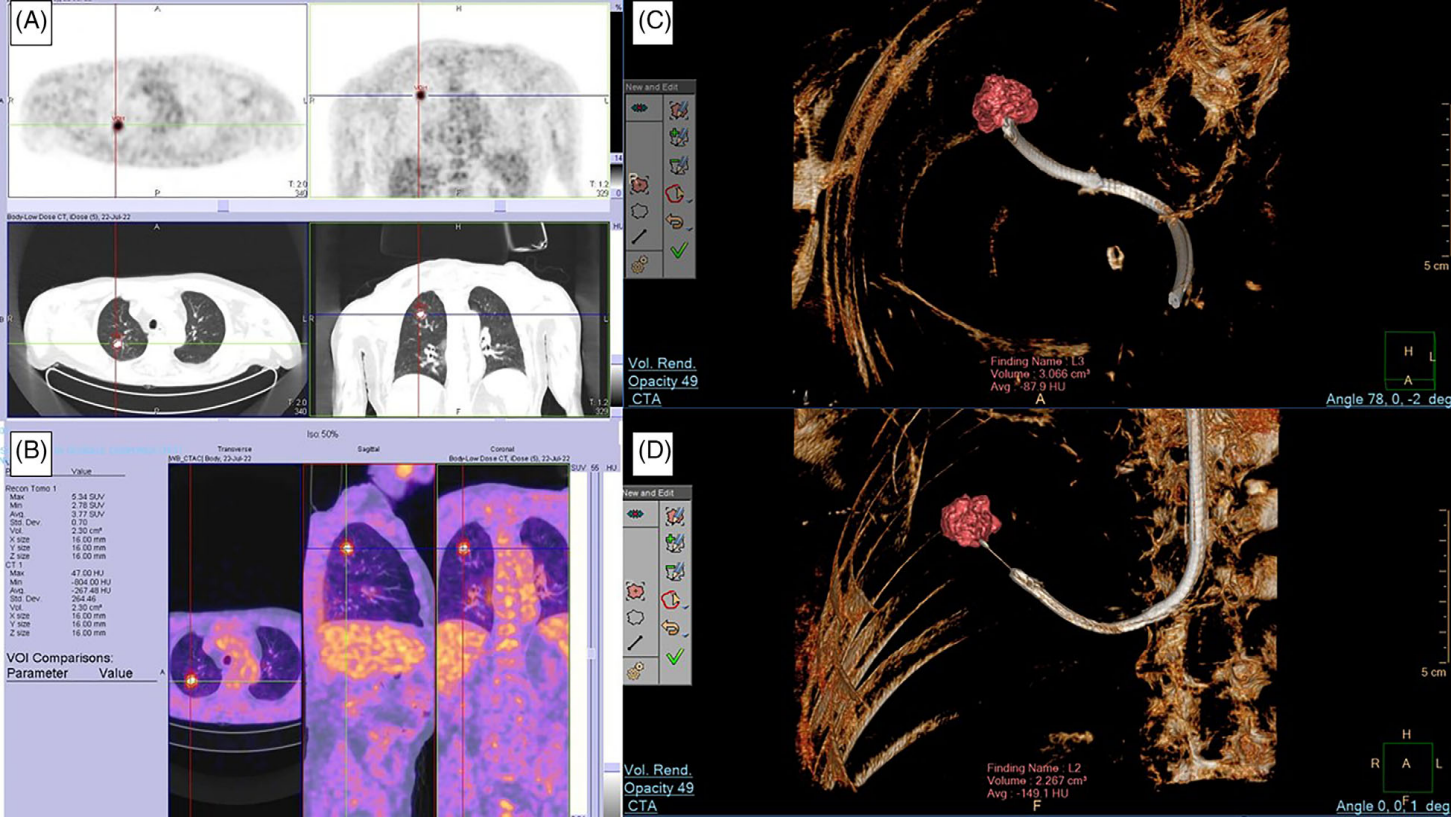
(A and B) 18F‐fluorodeoxyglucose (FDG) PET/CT uptake in correspondence of the right upper lobe; (B and C) aScope 5 Broncho HD 5.6/2.8 single‐use bronchoscope reach the lesion guided by 3D fluoroscopy CIOS guidance system: TBNA (C) and forceps (D) sampling (B).

A 73‐year‐old male, past smoker, came to our attention after the accidental finding of a pulmonary nodule located at the right upper lobe. The nodule characteristics were: dimensions 18 × 14 mm, not in contact with pleural surface, a bronchus sign type B, adjacent to the lesion. A 18F‐FDG PET was performed confirming the suspected malignancy nature of the lesion (Standardized Uptake Value max 5.34) without contrast uptake in correspondence with hilar lymph nodes (Figure 2A, B).

We performed bronchoscopy under general anaesthesia in laryngeal mask. We used aScope 5 Broncho HD 5.6/2.8 single‐use bronchoscope: after the confirmation of absence of endoscopically visible lesions, we proceeded with peripheral sampling with CIOS system. We introduced the 21‐Gauge TBNA tool through right B1 bronchus and, after the confirmation of the correct position of the needle into the lesion, we proceeded with the sampling. ROSE confirmed the adequacy of the sample and so we completed the sampling with another 2 passes with TBNA and 4 with biopsy forceps (Figure 2C, D). No procedure‐related complications were recorded. Both TBNA and biopsy forceps were easily introduced through the bronchoscope working channel without problems of manoeuvrability and the instrument was correctly operating after the procedure. Total acquisition time and KAP were 530 s and 72.1 Gycm^2^. Final diagnosis was adenocarcinoma and the material was adequate for molecular analysis.

## DISCUSSION

These cases focus our attention on the use of SUBs in sampling PPLs under fluoroscopic guidance; each instrument used gave us some advantages.

CIOS Mobile 3D spin system generates a CT image that allows the operator to confirm the tool‐in‐lesion during the sampling procedure, with higher rates if it is combined with other guidance instruments. Some recent studies demonstrate that the radiation dosage emitted by CIOS is similar to Cone Beam CT (CBCT) but, after the acquisition of expertise, this would decrease. In both our cases, the CIOS system allowed us to visualize the sampling tool in direct proximity to the lesion, and ROSE confirmed the adequacy of specimens. Although there is a brief latency between the acquisition (30 s) and elaboration (about 10 s) of images, the CIOS system gives us a 3D image of the bronchial tree and defines the relationship between lesion and sampling tool instrument. This is the main advantage with respect to conventional fluoroscopic guidance systems that, even if C‐arm would be rotated around the patient, giving us multiplanar prospective, the quality of images is lower.

As interventional pulmonologist we were familiar with RBs especially for their robustness that permits to stress the bronchoscopes during the sampling with tools inside, by rotating and bending them, passing through endotracheal tubes. Thin and ultrathin bronchoscopes overcome the limitation to achieve very distal lesions and bringing to the periphery of the lung thinner and more flexible 21‐Ga needles and cryoprobes.^1^ Nevertheless, the availability of ultrathin bronchoscopes is limited. Moreover, repair costs of RBs are not negligible: Rozman et al. calculated a repair cost of 5.25€ per procedure. The majority of the damages reported were located to the rubber coat on the distal bending section (59%), mainly caused by the repeated stress on the instruments' materials during procedures.^5^ The extreme bending generates a great mechanical stress of the angulation control lever and working channel. Furthermore, the introduction of needles and forceps increases the rigidity of the distal bending section, especially when the instrument is bended to reach lesions located in upper lobes. New generation Ambu® aScope™ 5 offers two distal diameters (5.6 and 5.0 mm) with different working channels (2.8 and 2.2 mm), improving the distal tip angulation range and flexibility. This increased manoeuvrability, in addition to a greater robustness of the entire bronchoscope, makes it more suitable for stressing procedures such as the sampling of peripheral nodules. Moreover, the diameter of the working channel is fit for an easy insertion of all sampling instruments (forceps and TBNA needles).

Other factors supported the introduction of SUBs in the sampling of PPLs. The portability of the system, consisting of the instrument and full‐HD monitor, allows examinations to be performed in hybrid operating rooms or other suites where guidance systems are present, since most bronchoscopy suites are not equipped with advanced medical imaging devices. This also allows examinations to be performed during surgery. Any possible damage caused by the sampling procedure would not let us without instrument for the subsequent procedure. From an organizational and economic perspective, SUBs are a better solution than a RBs in 75% of cases.^6^ From a clinical point of view, SUBs have many advantages, mainly the reduction of patient‐to‐patient infection transmission and personnel exposure to fomites or aerosol. However, considering the costs of a procedure performed with a reusable instrument varies from 355€ to 71€, and those using a SUBs is fixed at 232€, in a hospital performing less than 328 procedures per year, the use of SUBs becomes economically viable.^5^ Nevertheless, in a hospital with a large volume of procedures, the two solutions could be both valid and accessible, choosing the instrument on the basis of the indication.

In endobronchially invisible PPLs, the 3D fluoroscopy systems allow the operator to confirm the correct position of the sampling tools. Although SUBs are not routinely being used for advanced diagnostic bronchoscopy, the aScope 5 Broncho performed comparably to a reusable bronchoscope in terms of manoeuvrability with and without instruments in the working channel. aScope 5 Broncho may be a viable alternative to RBs for advanced diagnostic bronchoscopy.

## AUTHOR CONTRIBUTIONS

The authors equally contributed to the present work.

## CONFLICT OF INTEREST STATEMENT

None declared.

## ETHICS STATEMENT

The authors declare that appropriate written informed consent was obtained for the publication of this manuscript and accompanying images.

## Data Availability

The data that support the findings of this study are available on request from the corresponding author. The data are not publicly available due to privacy.
